# The New Sanatorium at Heatherside

**Published:** 1904-07-02

**Authors:** Edwin T. Kale


### THE NEW SANATORIUM AT HEATHERSIDE.

*To the Editor of* The Hospital.

Sir,—I have read with interest your account of our Sanatorium at Heatherside in your last issue. I note you mention that the sanatorium is for 88 patients. I thought you would like me to mention that it is for 100.

Yours faithfully,
Edwin T. Kale.

[∴ Our estimate of 88 beds erred owing to our having overlooked the intention to utilise as three-bedded wards the two compartments on either side of the day-room. In our opinion these rooms are ill-suited for phthisical patients, owing to their cramped situation and the absence of perfect ventilation.—Ed. Hospital.]

